# Mice from lines selectively bred for voluntary exercise are not more resistant to muscle injury caused by either contusion or wheel running

**DOI:** 10.1371/journal.pone.0278186

**Published:** 2022-11-30

**Authors:** Jarren C. Kay, James Colbath, Robert J. Talmadge, Theodore Garland

**Affiliations:** 1 Department of Evolution, Ecology and Organismal Biology, University of California, Riverside, CA, United States of America; 2 Department of Biological Sciences, California State Polytechnic University, Pomona, CA, United States of America; Bangor University, UNITED KINGDOM

## Abstract

Muscle injury can be caused by strenuous exercise, repetitive tasks or external forces. Populations that have experienced selection for high locomotor activity may have evolutionary adaptations that resist exercise-induced injury and/or enhance the ability to cope with injury. We tested this hypothesis with an experiment in which mice are bred for high voluntary wheel running. Mice from four high runner lines run ~three times more daily distance than those from four non-selected control lines. To test recovery from injury by external forces, mice experienced contusion via weight drop on the calf. After injury, running distance and speed were reduced in high runner but not control lines, suggesting that the ability of control mice to run exceeds their motivation. To test effects of injury from exercise, mice were housed with/without wheels for six days, then trunk blood was collected and muscles evaluated for injury and regeneration. Both high runner and control mice with wheels had increased histological indicators of injury in the soleus, and increased indicators of regeneration in the plantaris. High runner mice had relatively more central nuclei (regeneration indicator) than control in the soleus, regardless of wheel access. The subset of high runner mice with the mini-muscle phenotype (characterized by greatly reduced muscle mass and type IIb fibers) had lower plasma creatine kinase (indicator of muscle injury), more markers of injury in the deep gastrocnemius, and more markers of regeneration in the deep and superficial gastrocnemius than normal-muscled individuals. Contrary to our expectations, high runner mice were not more resistant to either type of injury.

## Introduction

The ability to locomote is essential to mammalian survival. Locomotion enables such activities as finding food or mates, defending territories, and migration. Often, these activities require mammals to travel large distances or at high speeds over varied terrain, which has an inherent risk of injury, including muscle injury. Muscle injuries are caused by a variety of factors, including (but not limited to) blunt trauma from external forces and overuse of the muscle itself. The resulting negative effects on locomotor capacity from muscle injury can severely compromise the ability of an animal to engage in various locomotor behaviors.

Although data on the frequency of muscle injuries in wild animals are not available, blunt trauma is a common mechanism for muscle injury (contusion) in human athletics [1–4]. In addition, muscle contusion is frequently studied in animal models [see 4 for review]. In male lab rats (*Rattus norvegicus*), a moderate contusion injury to the calf reduces maximum tetanic tension by 38% in an *in situ* preparation of the gastrocnemius complex on the day of injury and by ~20% after one week [5]. Contusion injuries to muscle are associated with increased pain (in humans), both at the site of injury and during movement [2,4], which could lead to reductions in locomotor performance beyond the effects on muscle contractile performance.

In addition to contusions, contractile activity itself can injure muscles. Over-exercising, such as running a marathon or sprinting both generally a mix of concentric and eccentric contractions; [6,7], or running downhill [only eccentric contractions; i.e., 8], will result in some degree of muscle injury, even in trained individuals. Eccentric (or lengthening) contractions produce the most injury [9], while concentric (or shortening) contractions will produce some injury, especially when performed over extended periods or to exhaustion [i.e. 10]. As with contusion injuries, data for exercise-induced muscle injury (of either type) in the wild is lacking, although the possibility that wild animals train via locomotor activity has recently been considered [11,12].

Surprisingly, even a moderate/routine amount of voluntary exercise has been shown to cause muscle injury, which is related to the amount of eccentric contractions performed [13]. In rats, downhill walking on a treadmill, which emphasized eccentric contraction of a knee extensor (vastus intermedius), for 26 bouts, five minutes each, at 15 m/min caused injury to the vastus intermedius [14].

Wheel running is a common model of exercise used in rodent studies of muscle function and likely consists of both concentric and eccentric muscle contractions. Mice appear to run both downhill and uphill during the same wheel running bout, especially in relatively large wheels [see video that accompanies 15]. Voluntary wheel running for as little as five days has been shown to induce muscle injury (measured histologically) in the soleus of two mouse strains [16]. On longer time scales (12 days to three months), no further acute injury was observed, but regeneration and satellite cells were common [16], suggesting some degree of recovery and/or the resistance to further injury.

Although exercise-induced injury is common even in trained individuals, a number of factors may influence susceptibility to this type of injury. For instance, rodents and humans that have previous training show less injury than those that are experiencing an exercise for the first time [17, see 18 for review]. This protection from injury may involve the expression of heat shock proteins, which may assist in providing protection from the mechanical stresses and increases in ROS that occur during exhaustive exercise [19].

Given that individuals may have different susceptibilities to injury, and that training state may have a protective effect against muscle injury, animals that have evolved to differ in the frequency or intensity of exercise behavior might be expected to differ also in the extent to which they resist exercise-induced injury and/or their ability to recover (rapidly) from such injury. The purpose of this study was to test this proposition in replicate lines of mice that had been selectively bred for more than 70 generations for high voluntary wheel running (High Runner or HR lines) as compared with non-selected control (C) lines. A number of exercise adaptations have been documented in HR mice, including increased maximal oxygen consumption (VO_2_max) and endurance during forced treadmill exercise, larger heart ventricles, more symmetrical hindlimb bones, and higher plasticity in GLUT4 transporters in gastrocnemius muscle when given access to wheels [20–25]; however, adaptations that may prevent injury or enhance the speed of recovery have not been studied.

One unexpected discovery in the selection experiment was the presence of a single nucleotide polymorphism (SNP) that, in homozygous individuals, causes a 50% reduction in hindlimb muscle mass, primarily due to greatly reduced MyHC-2b muscle fibers [both a reduction in size and number of fibers; 26–28]. The mutation has been identified as a SNP in an intron of the *Myh4* gene [29]. The general reduction of MyHC-2b fibers in the mini-muscle mouse are made up for by increases in MyHC-2x fibers and slight increases in MyHC-2a fibers [28]. The mini-muscle phenotype is associated with faster running speeds on wheels, increased cost of transport during voluntary wheel running, reduced maximal sprint speed, increased VO_2_max in some studies, larger soleus muscles, and medial gastrocnemius muscles that contract more slowly but are fatigue-resistant [28,30–33]. Additionally, mini-muscle mice have increased heat shock protein 72 (HSP72) concentrations in the triceps surae [34], which protects against exhaustive exercise-induced muscle injury in mice [e.g., see 35].

In this study, we examined the two types of injury discussed above. These protocols have their own sets of strengths and weaknesses. The contusion injury protocol ensures that each mouse receives the same level of injury; however, the mechanism of injury is different than that for exercise-induced injury. The voluntary wheel-running injury protocol does not cause the same level of injury in all mice (not all mice run the same amount), but it is more physiologically relevant to animals that have been bred for voluntary exercise. Additionally, it is difficult to disentangle injury caused by eccentric versus concentric contractions during wheel running as the mice in this study will have run both up- and downhill [see video that accompanies 15]. Therefore, we do not attempt to differentiate whether injury was caused by overuse or lengthening contractions during wheel running.

We hypothesized that HR mice have an innate ability to resist injury and/or that their recovery from injury happens faster than in C mice. We also expected that mini-muscle individuals would have increased muscle injury or subsequent regeneration in the superficial region of the gastrocnemius, a muscle that is primarily MyHC-2b fibers in mice, due to the muscle being smaller but having similar forces acting upon it as the normal-muscled individuals. Additionally, we expected that mini-muscle mice would show reduced injury in other muscles due to the protective effect of increased HSP72 levels. To examine these hypotheses, we conducted two experiments. First, we studied recovery from contusion injury, using voluntary wheel running as a proxy for recovery. Second, we studied exercise-induced muscle injury that may occur during voluntary wheel running (indicated by circulating concentrations of creatine kinase), as well as resistance to or recovery from injury (indicated by muscle histology). We used the triceps surae muscle complex for histological analysis, examining individual muscles and regions of known fiber type differences within the same muscle.

## Methods

### Animals

Male and female mice were sampled from the 72^nd^ and 74^th^ generation of an ongoing artificial selection experiment in which mice have been bred for voluntary wheel running [36,37]. The founding population was 224 outbred Hsd:ICR mice (*Mus domesticus*). Four selected high runner (HR) lines are bred based on wheel revolutions/day on days five and six of a six-day trial, while four control (C) lines are bred without regard to running. No sibling mating is allowed. Mice were weaned at 21 days of age and housed with food (Harlan Teklad Laboratory Rodent Diet (W)-8604, Los Angeles, CA, USA) and water provided *ad libitum* and a 12:12 photoperiod. At ~six-eight weeks of age, mice are individually housed with wheels for six days and given *ad libitum* food and water. The cages were attached to Wahman-type activity wheels (1.12 m circumference, 10 cm wide, 35.7 cm diameter) interfaced to a computer that records revolutions in one-minute intervals. Mice from selected lines were then bred based on the mean number of revolutions from days five plus six. The anesthesia used was isoflurane, and all animals were euthanized via decapitation. All experimental conditions and protocols and were approved by the University of California, Riverside institutional animal care and use committee (20170022).

### Wheel running and home-cage activity

For generations 71 and 73 mice that were housed individually with access to wheels for the testing period, running was measured as the number of revolutions in one-minute intervals for 23 hours/day by an interfaced computer [37,38].

Home-cage activity [HCA; a measure of spontaneous physical activity; 39] was measured for mice from generation 73 using a passive infrared sensor housed in wire mesh attached to the inside of the cage. Infrared sensors record activity three times per second as binary variables (0 = no movement, 1 = movement) and these readings are averaged for every one-minute interval over the course of 23 hours by software designed by Dr. Mark Chappell [33,40–42]. Sensor sensitivity was used as a covariate in all analyses [40].

### Contusion injury

50 male mice from HR lines seven and eight (lab designations), which lack the mini-muscle phenotype (see Introduction) and C lines one and two (lab designations) were either injured by a weight drop on the right triceps surae, or left uninjured as a control. All mice were then given access to wheels for six days. We did not apply the contusion-injury protocol to individuals with the mini-muscle phenotype because of the greatly reduced size of their triceps surae muscles and hence a concern that bone injury could occur.

On the day of injury, mice were anesthetized and immediately placed in position for the weight drop, with methods modified from Ota et al. [43] and Crisco et al. [5]. We did not provide analgesics as that would have interfered with the amount of wheel running, which was used as the metric of injury and recovery. Mice had their right legs and ankles extended and positioned offset from the opening of the pipe, such that only the muscle would be impacted. An 11.93 g steel ball bearing of 1.3 cm diameter was dropped through a 1.125 m polyvinyl chloride pipe with inner diameter of 1.51 cm onto the right triceps surae of each mouse. Once the weight had impacted the leg, the mice were weighed and returned to their home-cages with attached wheels. Time of injury and time of first wheel access were recorded to determine latency to run. Uninjured mice were anesthetized and had the weight placed on their triceps surae but did not have it dropped onto the muscle. Six days after injury, mice were sacrificed while under anesthesia to check for possible bone injury. For this experiment, wheel-running behavior was used as a biomarker both for the effect of the contusion injury and for the rate and degree of recovery from that injury; hence, these two aspects cannot be separated.

### Exercise-induced muscle injury

For the study of exercise-induced injury, 108 male mice from generation 73 were used, representing all four HR lines and all four C lines. Prior to the study, mice had not had any access to running wheels. Sixty-four mice were given access to wheels for six days (same as the selection protocol), and the other 44 were housed in individual cages without wheels. We used more mice in the group given wheel access in case wheel malfunctions made some of the data unusable. As described in the previous section, mice were sacrificed after six days and right triceps surae were dissected and weighed, then placed on cork and frozen in isopentane chilled in liquid nitrogen. Left triceps surae were also dissected from one line that remains polymorphic for the mini-muscle phenotype (HR line six) to determine mini-muscle status based on the relation between muscle mass and body mass [44; see Introduction]. We only obtained useable solei (undamaged from dissection or freezing) from 54 of the mice in this study. Only one of 14 line six mice showed the mini-muscle phenotype.

### Plasma creatine kinase activity

Trunk blood was collected for mice in the exercise-induced injury group via decapitation and centrifuged at ^~^7400 g (12000 rpm) at 4°C for 10 minutes, then the plasma was collected and stored at -80°C until use. Creatine kinase activity of the plasma was measured using a colorimetric assay kit (Cat#: KA3766; Abnova, Taipei City, Taiwan).

### Histology

Muscles were stored at -80°C until sectioning, when the belly of the muscle was removed and placed on cork and refrozen in liquid nitrogen. Muscles were cross-sectioned at 10 μm using a CM3050 S cryostat (Leica Microsystems, Buffalo Grove, IL, USA) at -20°C and adhered to charged slides (Thermo Fisher Scientific, Chino, CA, USA). Cross-sections from the belly of the triceps surae complex were then stained using a Rapid-Chrome Frozen Section Staining Kit for hematoxylin and eosin (H&E; Cat #: 9990001; Thermo Fisher Scientific, Chino, CA, USA), using three cross-sections for each stain. Muscle cross-sections were viewed using an Olympus BX51 microscope (Waltham, MA, USA) and photos were taken of the plantaris, soleus, and the superficial and deep regions of the gastrocnemius (we did not attempt to differentiate between the medial and lateral gastrocnemius) using a Retiga 2000RV camera (QImaging, Surrey, BC, Canada) at 10X with QCapture software (QImaging, Surrey, BC, Canada). Photos of the same region of each muscle group were taken at the same magnification for each of the three cross-sections per individual.

### Evaluation of exercise-induced muscle regeneration and injury

**Fig 1:**
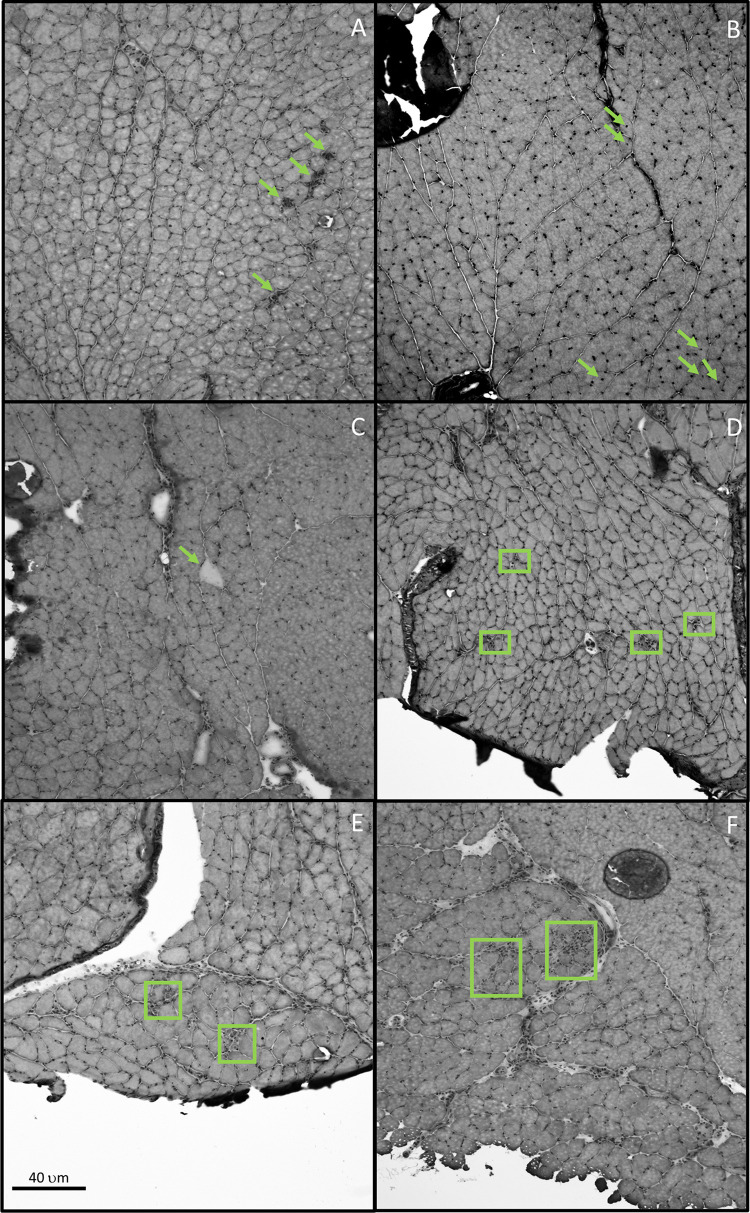
H&E staining of (A) myofibers in the deep gastrocnemius with invading cells (green arrows), (B) myofibers in the superficial gastrocnemius that exhibit centrally located nuclei (green arrows), (C) myofiber exhibiting pale staining cytoplasm in the deep gastrocnemius (green arrow), (D) areas in the plantaris showing signs of regeneration and do not contain central nuclei (green boxes), (E) soleus with necrotic fibers (green boxes), and (F) soleus with perimysial infiltration (green boxes). All representative pictures come from normal-muscled mice (not mini-muscle individuals) at 10x.

Images of hematoxylin and eosin (H&E) stained muscle cross-sections were analyzed using Image J software (U.S. National Institutes of Health, Bethesda, MD, USA). The digital images were evaluated for muscle fiber injury and regeneration using a modified criteria from Tsivitse et al [45,46]. Specifically, areas considered as regenerating included the following: centrally located nuclei (counted as any nuclei not touching the sarcolemma, i.e., directly adjacent to the endomysium, and not containing any other metric of injury/regeneration), and areas of regeneration that did not include central nuclei. Cells exhibiting pale cytoplasm, obvious signs of necrosis (broken or degrading cells within a myofiber), and myofibers that were invaded by mononuclear cells were considered injured (see Fig 1). An additional metric was also used as an indicator of injury: regions of muscle that included infiltration by mononuclear cells into the perimysium but not into the myofibers themselves (Fig 1F). This metric was ranked on a 0–3 scale (0 having no evidence of infiltration and 3 having extensive infiltration). Perimysial infiltration was ranked blind on two separate occasions by the same individual. If those ranks did not match, they were blind ranked a third time and the closest ranks were averaged together and used for statistical analysis. The three cross-sections were counted for markers of muscle injury/regeneration and those were then summed across the three cross-sections. Values are presented as the percent of the total number of myofibers that show one or more histological markers of injury.’

### Statistical analysis

We used the Mixed Procedure in SAS 9.4 (SAS Institute, Cary, NC, USA) to apply a nested analysis of covariance (ANCOVA) models, with replicate lines nested within linetype (HR vs. Control) as a random effect. In cases of zero-inflated data (i.e., many mice had values of zero), the Procedure GLIMMIX was used, again nesting line within linetype. In both cases, main effects were linetype and either injury status or wheel access. For experiment two, mini-muscle status was an additional main effect, and we also tested for mini * wheel access interactions. Age at dissection, standardized age at dissection squared (i.e., orthogonal polynomial), time of dissection, and standardized time of dissection^2^ were used as covariates in all analyses of injury or regeneration in experiment two. (Standardization refers to subtracting the mean and dividing by the standard deviation of a variable.) For wheel running (in both experiments), wheel freeness (an inverse measure of how difficult it is to turn the wheel) was used as an additional covariate [40].

In the contusion experiment (using only 2 HR and 2 C lines), the degrees of freedom for testing the effects of linetype and injury status were 1 and 2. In the exercise experiment (using all 4 HR and 4 C lines), the degrees of freedom for testing the effects of linetype or wheel access (training) were 1 and 6. Also in the exercise experiment, the mini factor and the mini * wheel access interaction were tested relative to the residual d.f. However, in the exercise experiment, if the wheel access * (line)linetype interaction covariance parameter estimate was zero, it was removed from the model and the effect of wheel access and wheel access * linetype were tested over the residual d.f. For both experiments, main effects were considered statistically significant at p < 0.05; interactions were considered significant at p < 0.1 because ANOVA models typically have reduced power to detect interactions as compared with main effects [34, e.g., see 47,48].

For total injury (combining all markers of injury), total regeneration (combining all markers of regeneration), and combined injury plus regeneration (combining all markers of injury and regeneration), in order to weight each component trait equally, we first standardized each component measure by subtracting the mean from the individual values and then dividing by the standard deviation. These standardized values were then summed to obtain the composite score. This procedure was followed because not all markers were presented as a percentage of the total number of fibers, and also because variances differed among measures. Additional transforms were done to the standardized variables to improve normality of the residuals. For total regeneration in the plantaris, it was necessary to rank-transform the standardized values to achieve normality of the residuals.

For individual injury markers that were zero-inflated, simplified 0/1 variables were made indicating whether any cells in that muscle cross-section contained the marker in question or not. Then a Z-test was used to look for differences in the proportions of injured and non-injured individuals among different groups (e.g. C vs HR) and to compare different types of injury markers between muscles (e.g. plantaris vs soleus).

## Results

### Effects of contusion injury on voluntary wheel-running behavior

**Fig 2:**
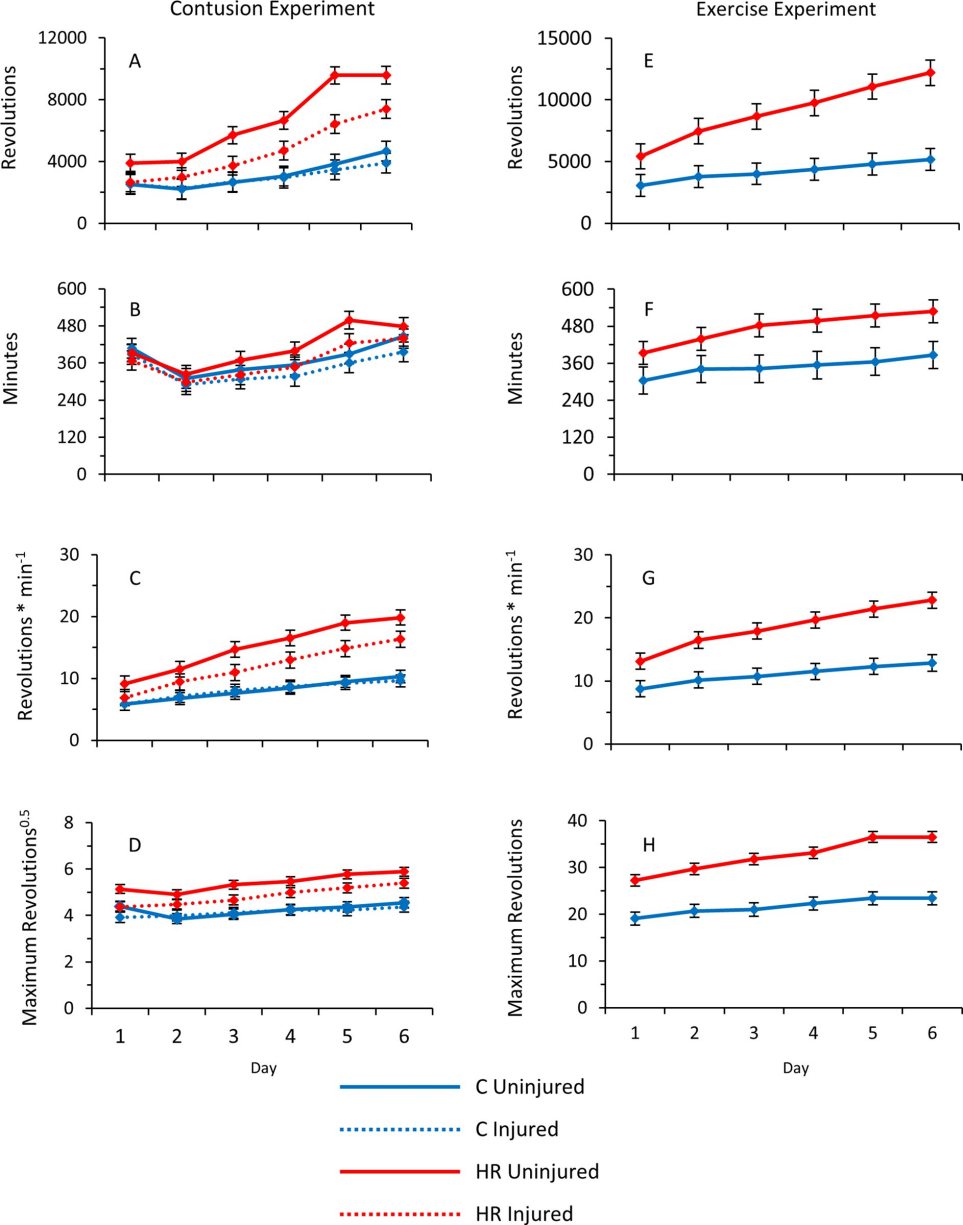
Average wheel running metrics across six days for the both the Contusion Experiment (A-D) and the Exercise Experiment (E-H) shown in Tables 3 and 4. (A) Total revolutions (n = 42). (B) Number of one-minute intervals with at least one revolution (running duration; n = 42). (C) Revolutions per minute (average speed; n = 42). (D) Maximum number of revolutions in any one-minute interval (n = 41). (E) Total revolutions (n = 61). (F) Revolutions per minute (average speed; n = 60). (G) Number of one-minute intervals with at least one revolution (running duration; n = 61). (H) Maximum number of revolutions in any one-minute interval (n = 61). Values are LS means ± standard errors from SAS Procedure Mixed repeated-measures ANCOVA.

Dissections indicated one individual with a fractured tibiafibula that was then excluded from all analyses. Given the known large difference in daily wheel running between HR and C mice [e.g., see 34], we first analyzed the two linetypes separately, treating the two replicate lines within each linetype as a fixed effect. All measures of wheel running generally increased across the 6-day trial, regardless of injury status, for both HR and C mice, although minutes run per day decreased from day 1 to 2 for all four groups (Table 1, Fig 2).

**Table 1:** Effects of contusion injury on voluntary wheel-running behavior, split by linetype (contusion experiment).

High Runner	RUN			INT			RPM			MAX		
**N =**		20			20			19			19	
	**d.f.**	**F**	**P**	**d.f.**	**F**	**P**	**d.f.**	**F**	**P**	**d.f.**	**F**	**P**
**P**_**Line**_	1, 15	0.25	0.6245+	1, 15	0.35	0.5647+	1, 14	6.23	**0.0257+**	1, 14	0.74	0.4041+
**P**_**Injury**_	1, 15	5.88	**0.0284-**	1, 15	1.45	0.2473-	1, 14	12.62	**0.0032-**	1, 14	9.28	**0.0087-**
**P**_**Day**_	1, 80	15.69	**< .0001**	1, 80	7.87	**< .0001**	1, 75	36.71	**< .0001**	1, 75	10.77	**< .0001**
**P**_Line*Injury_	1, 15	0.06	0.8150	1, 15	0.13	0.7250	1, 14	1.95	0.1840	1, 14	0.1	0.7576
**P**_Line*Day_	1, 80	0.67	0.6466	1, 80	0.18	0.9678	1, 75	2.91	**0.0188**	1, 75	0.53	0.7503
**P**_Injury*Day_	1, 80	1.03	0.4083	1, 80	0.32	0.9019	1, 75	1.24	0.3004	1, 75	1.18	0.3265
**P**_Line*Injury*Day_	1, 80	0.37	0.8663	1, 80	0.53	0.7524	1, 75	1.05	0.3957	1, 75	0.95	0.4523
**P**_**Wheel Freeness**_	1, 15	1.69	0.2138+	1, 15	0.99	0.3355+	1, 14	1.14	0.3036+	1, 14	3.24	0.0933+
**Control**	**RUN**			**INT**			**RPM**			**MAX**		
**N =**		22			22			21			20	
	**d.f.**	**F**	**P**	**d.f.**	**F**	**P**	**d.f.**	**F**	**P**	**d.f.**	**F**	**P**
**P**_**Line**_	1, 17	2.03	0.1719+	1, 17	1.03	0.3240-	1, 16	1.15	0.2988+	1, 15	1.41	0.2537+
**P**_**Injury**_	1, 17	0.08	0.7874-	1, 17	1.01	0.3292-	1, 16	0.21	0.6496-	1, 15	0.02	0.9016-
**P**_**Day**_	5, 90	8.98	**< .0001**	5, 90	14.05	**< .0001**	1, 85	17.25	**< .0001**	1, 80	3.1	**0.0131**
**P**_Line*Injury_	1, 17	2.37	0.1424	1, 17	1.68	0.2122	1, 16	0.56	0.4643	1, 15	4.71	**0.0464**
**P**_Line*Day_	5, 90	0.58	0.7187	5, 90	2.08	**0.0749**	1, 85	1.74	0.1331	1, 80	2.55	**0.0341**
**P**_Injury*Day_	5, 90	0.46	0.8031	5, 90	0.26	0.9353	1, 85	1.39	0.2370	1, 80	3.23	**0.0104**
**P**_Line*Injury*Day_	5, 90	0.54	0.7476	5, 90	0.98	0.4365	1, 85	0.35	0.8791	1, 80	0.23	0.9479
**P**_**Wheel Freeness**_	1, 17	2.82	0.1115+	1, 17	5.55	**0.0308+**	1, 16	1.07	0.3156+	1, 15	3.11	0.0984+

P values from repeated measures ANCOVA analyzing wheel running traits across six days. Bold indicates significant differences (p < 0.05 or p < 0.10 for interactions). Positive (+) indicates direction Line 8 > Line 7, Line 2 > Line 1, and Injured > Uninjured. Wheel freeness was transformed to the 0.4 power to normalize residuals. RUN = total number of revolutions. INT = number of intervals with ≥ one revolution. RPM = revolutions per minute. MAX = maximum revolutions in any one-minute interval.

Mice from the two HR lines decreased revolutions run per day when injured (p = 0.0284; Fig 2A), which was attributable to decreased average and maximal speed (p = 0.0032 and p = 0.0087, respectively; Fig 2C and 2D), but not a decrease in running duration (p = 0.2473; Fig 2B and Table 1). As seen in a previous study [49], HR Line 7 mice ran faster than HR Line 8 mice (Table 1). Mice from the two Control lines had no significant effect of injury on any measure of wheel running (Table 1). In the C lines tested, maximum running speed shows a significant line * injury interaction with injured Line 2 mice attaining higher maximum speeds than injured Line 1 mice (p = 0.0464).

When analyzed together (Table 2), HR mice ran significantly more than C mice on all days, regardless of injury (p = 0.0414; Fig 2A), and this result was mirrored in their running speed and the maximum revolutions in any one-minute interval (square root transformed to improve normality of the residuals; p = 0.0484 and p = 0.0346, respectively; Fig 2C and 2D).

**Table 2:** Effects of contusion injury on voluntary wheel-running behavior, not split by linetype (contusion experiment).

	RUN			INT			RPM			MAX		
N =		42			42			42			41	
	**d.f.**	**F**	**P**	**d.f.**	**F**	**P**	**d.f.**	**F**	**P**	**d.f.**	**F**	**P**
**P**_**Linetype**_	1, 2	22.67	**0.0414+**	1, 2	1.47	0.3492+	1, 2	19.17	**0.0484+**	1, 2	27.41	**0.0346+**
**P**_**Injury**_	1, 2	4.83	0.1592-	1, 2	2.96	0.2277-	1, 2	4.14	0.1787-	1, 2	4.42	0.1704-
**P**_**Day**_	5, 10	24.62	**< .0001**	5, 10	14.74	**0.0002**	5, 10	39.59	**< .0001**	5, 10	6.34	**0.0067**
**P**_Linetype*Injury_	1, 2	3.24	0.2136	1, 2	0.09	0.7949	1, 2	4.22	0.1765	1, 2	2.17	0.2788
**P**_Linetype*Day_	5, 10	6.54	**0.0060**	5, 10	1.69	0.2247	5, 10	7.25	**0.0041**	5, 10	1.07	0.4327
**P**_Injury*Day_	5, 10	1.09	0.4234	5, 10	0.17	0.9698	5, 10	1.01	0.4595	5, 10	2.91	**0.0707**
**P**_Linetype*Injury*Day_	5, 10	0.98	0.4742	5, 10	0.35	0.8690	5, 10	0.97	0.4825	5, 10	0.48	0.7867
**P**_**Wheel Freeness**_	1, 203	4.84	**0.0289+**	1, 203	7.22	**0.0078+**	1, 203	2.23	0.1372+	1, 197	3.92	**0.0490+**

For explanation, see footnotes for Table 1.

### Exercise-induced injury

#### Wheel running

Mice from the four replicate HR lines ran more total revolutions than those from the four C lines on all days, and the differential became larger across the 6 days of wheel access (linetype * day interaction p = 0.0001; Fig 2E and Table 3). HR mice increased their wheel running from ~5,400 revolutions on day 1 to ~12,200 revolutions on day 6, as compared with C mice which increased from ~3,000 revolutions to ~5,200 revolutions during the same span (Fig 2E). The higher daily running distances of HR mice were caused by greater duration of running (Fig 2F), and greater average running speeds (Fig 2G [maximum speeds were also higher in HR mice Fig 2H]). The increasing differential in daily running distances was attributable to an increasing disparity in running speeds (significant linetype * day interactions), not duration (interaction P = 0.3875; Table 3). As compared with normal-muscled individuals, mini-muscle mice ran significantly faster and had a higher maximum number of revolutions in any one-minute interval (Table 3).

**Table 3:** Comparisons of voluntary wheel-running behavior between high runner and control lines of mice during the exercise-induced injury study (exercise experiment).

		RUN			INT			RPM			MAX		
N			61			60			61			61	
		d.f.	F	P	d.f.	F	P	d.f.	F	P	d.f.	F	P
**P**_**Linetype**_		1, 6	20.27	**0.0041+**	1, 6	6.79	**0.0404+**	1, 6	26.30	**0.0022+**	1, 6	58.17	**0.0003+**
**P**_**Day**_		5, 30	27.32	**< .0001**	5, 30	8.55	**< .0001**	5, 30	49.92	**< .0001**	5, 30	19.41	**< .0001**
**P**_Linetype*Day_		5, 30	7.46	**0.0001**	5, 30	1.09	0.3875	5, 30	8.21	**< .0001**	5, 30	3.09	**0.0228**
**P**_**Mini**_		1, 313	1.41	0.2356+	1, 307	0.40	0.5269-	1, 313	4.66	**0.0317+**	1, 313	8.28	**0.0043+**
**P**_**TWheel Freeness**_		1, 313	1.80	0.1803+	1, 307	4.39	**0.0369+**	1, 313	0.01	0.9158+	1, 313	0.17	0.6770+

Significance levels (p values) from repeated-measures ANCOVA analyzing wheel running parameters across six days. Bold values indicate significant differences (p < 0.05 or p < 0.10 for interactions). Positive (+) indicates direction HR > C and Mini > Normal. Wheel freeness was transformed 0.5 power to normalize the distribution of residuals. RUN = total number of revolutions. INT = number of intervals with at least one revolution. RPM = revolutions per minute. MAX = maximum number of revolutions in any one-minute interval.

#### Home-cage activity

**Fig 3:**
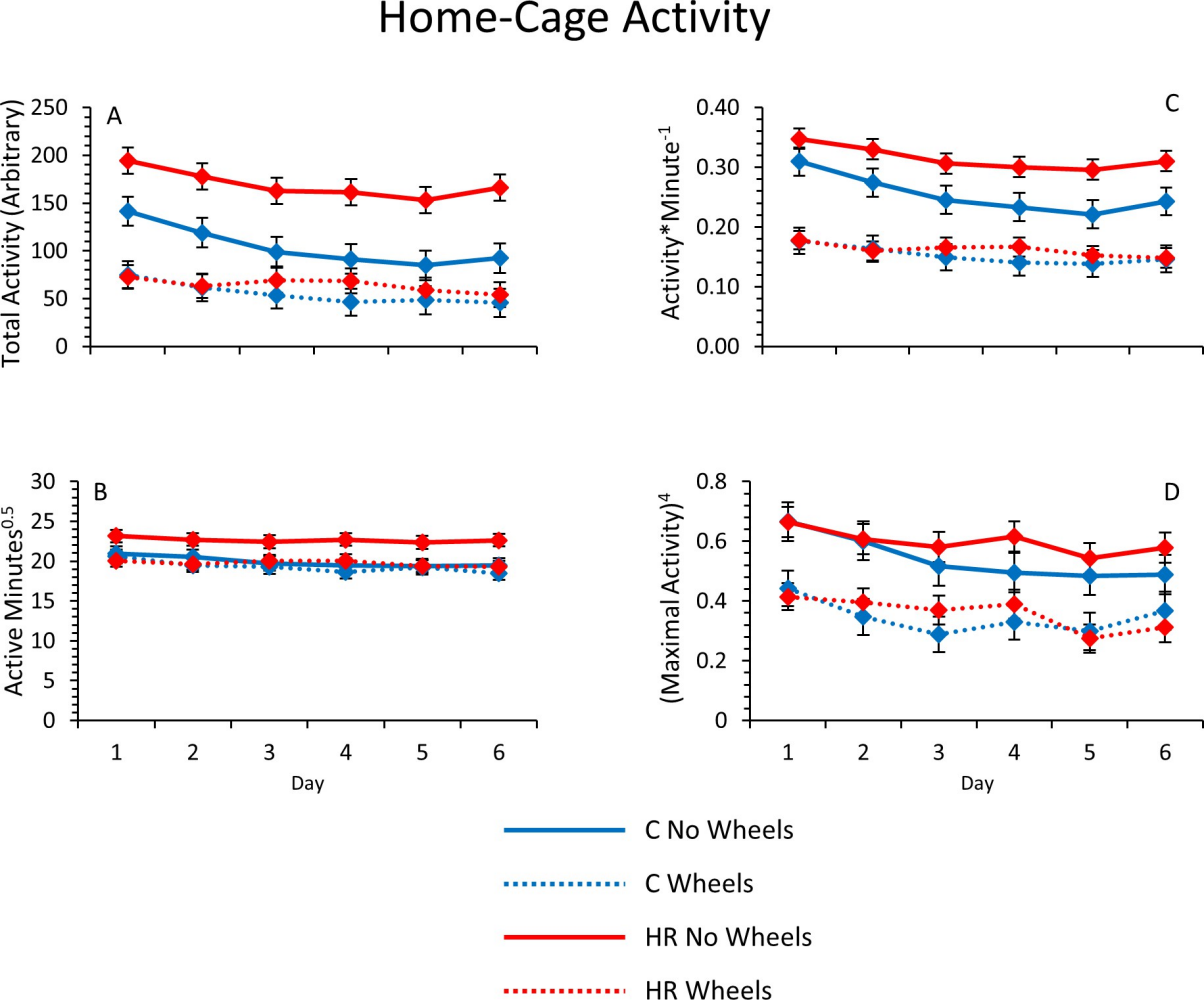
Average home-cage activity metrics across six days for male HR and C mice without and with access to wheels (exercise experiment). (A) Total HCA (n = 86). (B) Time spent active (square root transformed; n = 86). (C) Mean activity per minute (n = 86). (D) Mean maximum activity in any one-minute interval (transformed to the 4.0 power; n = 86). Values are LS means ± standard errors from SAS Procedure Mixed repeated-measures ANCOVA.

As seen in previous studies, all HCA measures decreased across the 6 day trial in all mice [41; Fig 3]. Mice with wheel access were less active in their cages than mice without wheels (p = 0.0003), and this effect was greater in HR lines (linetype*wheel access p = 0.0373; Fig 3A). Mice with wheel access were also active for less total time, had lower activity per minute, and lower maximum activity in any one-minute interval than mice housed without wheels (p = 0.0346, p = 0.0002, and p = 0.0012, respectively; Fig 3B–3D, respectively). In general, HR mice were more active (p = 0.0259 for total activity) and tended to be active for more minutes per day and at a higher average intensity of activity (p = 0.0735 and p = 0.0901, respectively). Mini-muscle status had no significant effects on any measure of HCA.

#### Plasma creatine kinase activity

Mini-muscle mice had lower plasma creatine kinase activity (an indicator of muscle injury) than normal-muscled mice (LSmeans = 0.012 ± 0.0017 and 0.018 ± 0.0006 for mini-muscle and normal-muscle respectively; p = 0.0033; Table 4), with no overall effects of linetype (p = 0.2249) or wheel access (p = 0.5134), and no significant interactions. Adding the indicators of home-cage activity (averaged over days 5 and 6 or all 6 days) as covariates (one at a time) indicated that they were not significant predictors of creatine kinase activity and had little effect on the significance levels for the main effects (results not shown). In a separate analysis of only mice that had wheel access, mini-muscle individuals again had significantly lower plasma creatine kinase activity than normal-muscled mice (p = 0.0144). Adding the wheel-running metrics or the home-cage metrics (averaged over days 5 and 6 or all 6 days) to this analysis indicated that none of them was a significant predictor of plasma creatine kinase, and the mini-muscle effect always remained significant.

**Table 4:** Comparisons of plasma and histological indicators of exercise-induced muscle injury in the triceps surae of HR and C mice (exercise experiment).

Trait	N	Transform	P_Linetype_	P_Wheel Access_	P_Linetype*Wheel Access_	P_Mini_	P_Mini*Wheel Access_
Creatine Kinase Activity	106	-	0.2249+	0.5134-	0.3572	0.0033-	0.6502
Total Injury							
Deep Gastrocnemius	92	(5 + ZINJDG)**0.01	0.3917-	0.3223+	0.8505	**0.0127+**	0.8822
Superficial Gastrocnemius^#^	89	-	-	-	-	-	-
Plantaris	93	(5 + ZINJPL)**0.02	0.6543+	0.8879+	0.1478	0.2817+	0.4726
Soleus	51	(5 + ZINJSL)**0.2	0.8524+	**0.0114+**	0.6181	0.8769+	0.1353
% Central Nuclei							
Deep Gastrocnemius	91	1+LOG10	0.1718+	0.6329-	0.1108	**0.0296+**	0.7508
Superficial Gastrocnemius	86	1+LOG10	0.8240+	0.5859+	0.8841	**< .0001+**	**0.0331**
Plantaris	93	1+LOG10	0.1831+	0.0525+	0.3246	**0.0454+**	0.3532
Soleus	52	1+LOG10	**0.0193+**	0.2453-	0.6029	0.9217+	**0.0975**
Total Regeneration							
Deep Gastrocnemius	91	(5 + ZREGDG)**0.2	0.4949+	0.5618-	**0.0764**	**0.0072+**	0.9247
Superficial Gastrocnemius^‡^	89	10 + ZREGSG	0.9888+	0.9631+	0.9126	**0.0271+**	0.8891
Plantaris	94	STANDARDIZED AND RANK	0.6055+	**0.0449+**	0.5965	0.8545+	**0.0850**
Soleus	51	STANDARDIZED	0.6146-	0.2443-	0.8190	0.7666+	**0.0972**
							

^#^The number of individuals without any marker of injury was too high to allow for a normal distribution and was therefore not analyzed. ^‡^The GLIMMIX procedure (SAS) was used here to give a normal distribution of the data.

Significance levels (p values) from ANCOVA. Bold values indicate significant differences (p < 0.05 or p < 0.10 for interactions). Positive (+) indicates direction Wheel Access > No Wheel Access, HR > C and Mini > Normal. Total Injury = number of cells exhibiting standardized necrotic fibers, standardized perimysial infiltration, standardized invaded fibers, and/or standardized pale staining cytoplasm. Total Regeneration = number of cells exhibiting either standardized central nuclei and/or standardized areas of regeneration. Additional analyses included the metrics of wheel running or of home-cage activity as covariates (see text).

When analyzing only mice that did not have access to wheels, mini-muscle individuals tended to have lower plasma creatine kinase compared to normal-muscled mice (p = 0.0619). Adding the home-cage metrics to this analysis indicated that none of them was a significant predictor of plasma creatine kinase, and the mini-muscle effect remained similar.

#### Total muscle injury

For the index of total muscle injury, the superficial gastrocnemius showed very few signs of injury and was heavily zero inflated (many individuals showed none of the indicators of muscle injury used in this study); hence, this muscle could not be analyzed statistically (models did not converge). The soleus showed a statistically significant increase in the index of total muscle injury with wheel access (p = 0.0144; Table 4), with no effect of linetype and no interactions. Effects of wheel access, linetype, and their interaction were non-significant for the deep gastrocnemius and plantaris. In the deep gastrocnemius, mini-muscle mice had more total injury than other mice, regardless of wheel access (p = 0.0127; Table 4). Adding the indicators of home-cage activity as covariates, one at a time, indicated that none of them were significant predictors of total injury for any muscle, and they had little effect on significance levels of the other factors (results not shown).

In a separate analysis using only mice that had wheel access, mini-muscle individuals had significantly more injury than normal-muscled mice in the deep gastrocnemius (p = 0.0149). Adding the various measures of wheel running (averaged across all 6 days or only the first 3 days) individually as covariates never had significant effects on the total amount of injury for any muscle, and they caused little change in the significance levels for linetype or mini-muscle status. When mice without wheel access were analyzed separately, we found no significant effects of linetype or mini-muscle status on total injury for any muscle (results not shown), and adding the home-cage metrics to this analysis indicated that none of them were significant predictors of total injury.

#### Central nuclei

**Fig 4:**
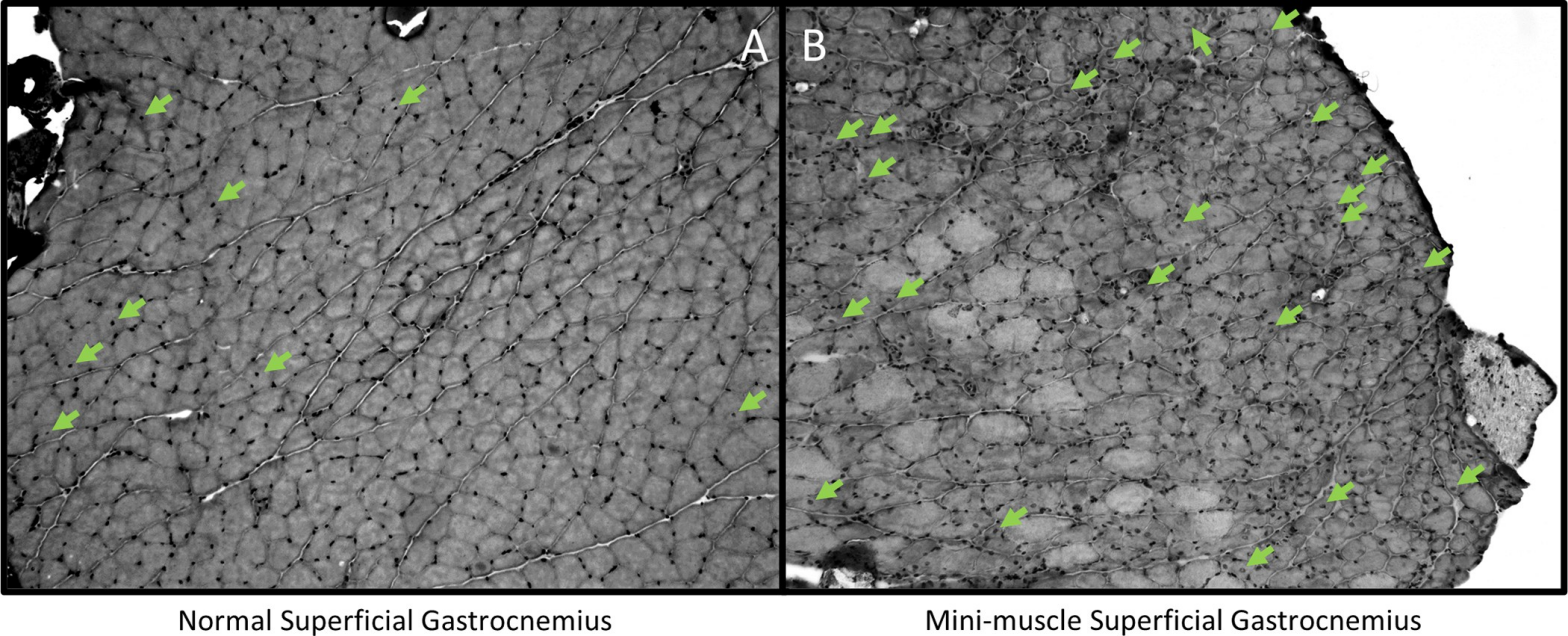
Percentage of fibers that contained centrally located nuclei in different muscles (exercise experiment). (A) C vs HR mice with and without wheel access. (B) Normal vs mini-muscle individuals with or without wheel access. Values are 1 + log_10_ transformed LS means ± standard errors from SAS Procedure Mixed ANCOVA. DG = deep gastrocnemius, SG = superficial gastrocnemius, PL = plantaris, SL = soleus.

**Fig 5:**
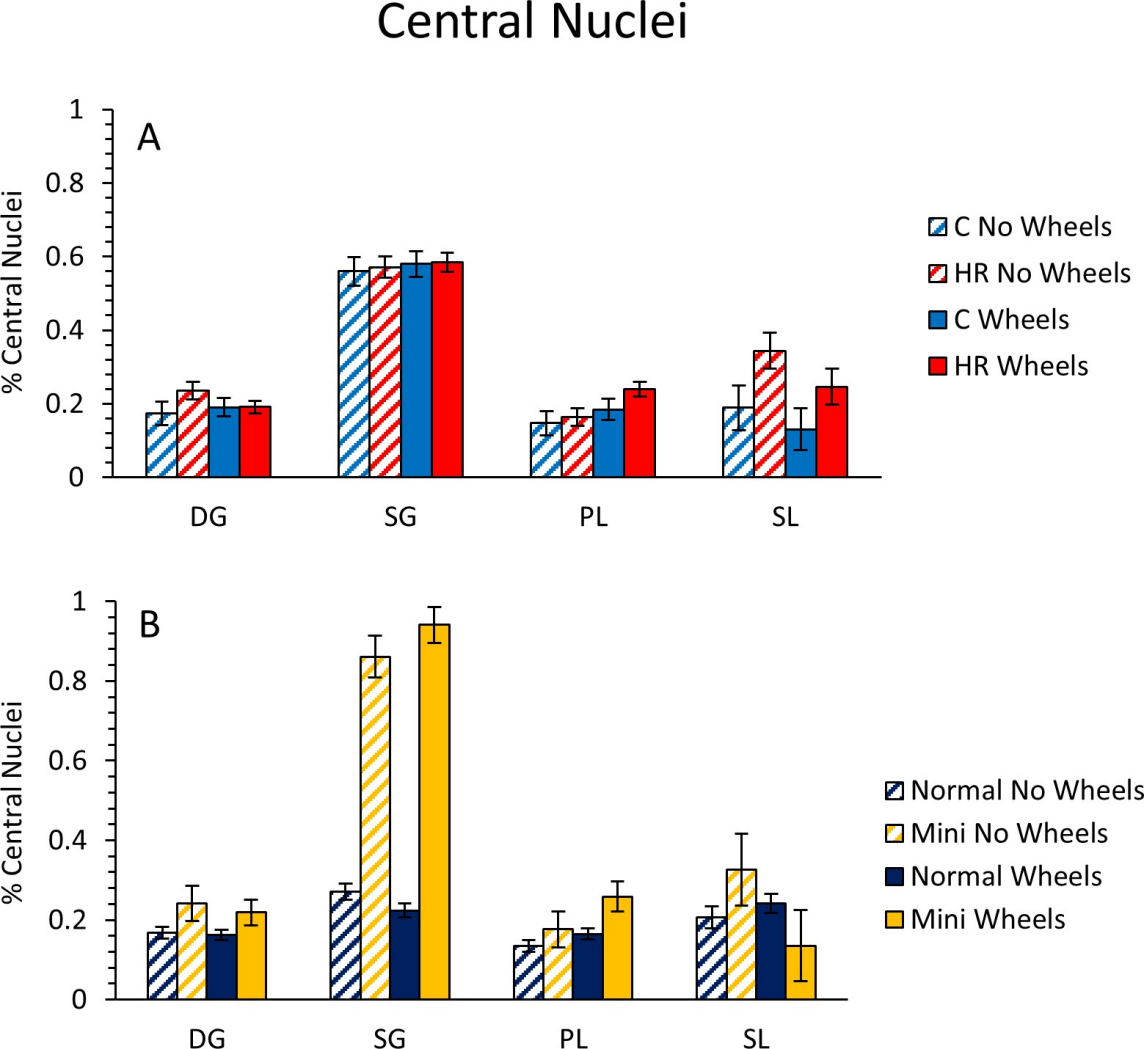
H&E staining of a normal-muscled superficial gastrocnemius and a mini-muscle superficial gastrocnemius taken at 10x. Green arrows indicate centrally located nuclei. These individuals had no wheel access.

Central nuclei are used as a biomarker of regeneration. In this study, the major observed differences in the percentage of central nuclei involved mini-muscle individuals. In the deep gastrocnemius, mini-muscle individuals had a higher percentage of fibers with central nuclei than normal-muscled individuals (p = 0.0296; Fig 4B). In the superficial gastrocnemius, mini-muscle individuals also had a higher percentage of fibers with central nuclei (p < 0.0001; Figs 4B and 5; Table 4), and wheel access increased this percentage, whereas it decreased the percentage in normal-muscled individuals (mini-muscle*wheel access interaction p = 0.0331; Table 4). In the plantaris, mini-muscle mice again had a significantly higher percentage of fibers with central nuclei (p = 0.0454; Fig 4B), and mice with wheel access tended to have a higher percentage of fibers with central nuclei than those without wheels (p = 0.0525; Table 4). In the soleus, HR mice had a higher percentage of fibers with central nuclei than C mice (p = 0.0193; Fig 4A). Wheel access reduced the percentage of fibers with central nuclei in mini-muscle mice, but tended to increase it in normal-muscled individuals (mini-muscle*wheel access interaction p = 0.0975; Fig 4B). Adding the indicators of home-cage activity as covariates, one at a time, indicated that none of them were significant predictors of percent central nuclei for any muscle, and they had little effect on significance levels of the other factors (results not shown).

Considering only mice with wheels, mini-muscle individuals had a higher percentage of fibers with central nuclei in the superficial gastrocnemius and the plantaris than normal-muscled mice (p < 0.0001 and p = 0.0218, respectively; results not shown in a table), with a trend for the soleus of HR mice to have an increased percentage of fibers with central nuclei than C mice (p = 0.0749). In models with the average amount of wheel running on days 1–6 as a covariate, this metric negatively predicted the percentage of central nuclei in the plantaris (p = 0.0490; results not shown in a table) and HR and mini-muscle mice had a higher percentage of fibers with central nuclei (linetype p = 0.0243 and mini p = 0.0099). Similar results were found when adding the average speed at which mice ran on days 1 through 6 (covariate p = 0.0149; results not shown in a table). Using the average amount of wheel running, average speed or average time spent running on days 1–3 as covariates had similar effects as days 1–6 (results not shown).

When mice without wheels were analyzed separately, mini-muscle individuals had a higher percentage of fibers containing central nuclei in the deep and superficial gastrocnemius (p = 0.0108 and p <0.0001, respectively; results not shown in a table), as compared with normal-muscled mice.

#### Total muscle regeneration

The total amount of regeneration (the number of central nuclei and areas of regeneration combined) was higher in the deep and superficial gastrocnemius of mini-muscle mice as compared with normal-muscled mice (p = 0.0072 and p = 0.0271, respectively; Table 4). Also in the deep gastrocnemius, a linetype * wheel access interaction showed that mice from HR lines had less regeneration after wheel access, whereas mice from C lines had more regeneration after wheel access (interaction p = 0.0764; Table 4). Regeneration was also higher in the plantaris of mice that had wheel access as compared with those not allowed access to running wheels (p = 0.0449; Table 4). In the plantaris, mini-muscle mice had a greater increase in regeneration after wheel access than did normal-muscled mice (mini * wheel access interaction p = 0.0850; Table 4). In the soleus, mini-muscle individuals had less regeneration after wheel access, whereas normal-muscled mice had more (mini * wheel access interaction p = 0.0972; Table 4). Adding the indicators of home-cage activity as covariates, one at a time, indicated that none of them were significant predictors of total regeneration for any muscle, and they had little effect on significance levels of the other factors (results not shown).

In a separate analysis of only mice that had wheel access, the deep gastrocnemius of mini-muscle mice had significantly more regeneration than normal-muscled mice (p = 0.0218), with a trend for the superficial gastrocnemius and the plantaris of mini-muscle individuals to have more regeneration (p = 0.0627 and p = 0.0834, respectively). The average amount of time spent running on nights 1–6 tended to negatively predict the amount of regeneration in the plantaris (p = 0.0594). The average amount of time spent running during days 1–3 negatively predicted regeneration in the plantaris (p = 0.0200) and tended to negatively predict regeneration in the soleus (p = 0.0633). No other wheel-running measure had a significant effect on the total amount of regeneration (results not shown). When mice without wheel access were analyzed separately, the deep gastrocnemius of HR mice tended to have more regeneration than C mice (p = 0.0638), with no effect of the mini-muscle phenotype (p = 0.2011).

#### Comparisons of different muscle groups

**Fig 6:**
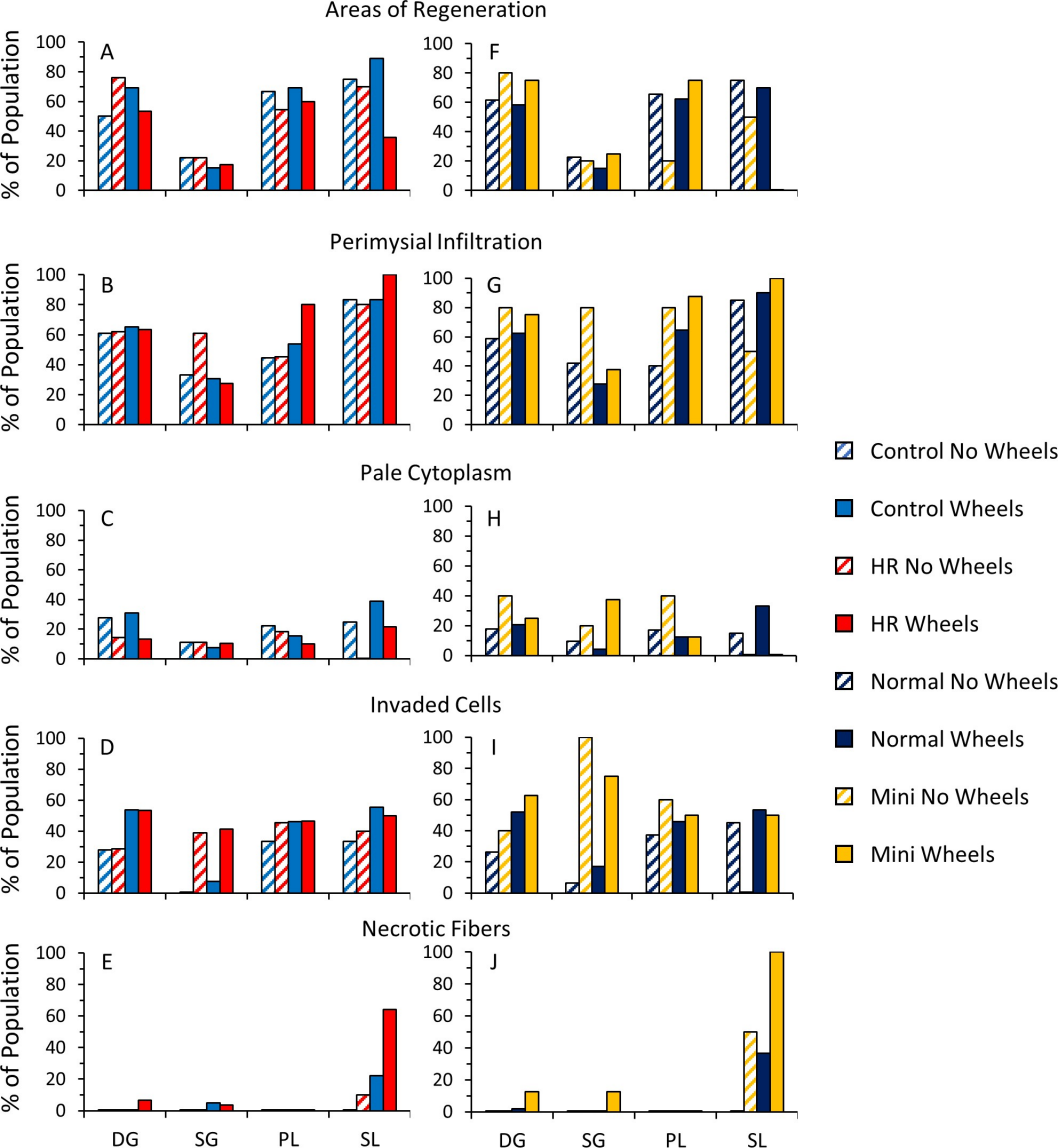
Proportion of the population of C and HR (A-E) and normal- and mini-muscled (F-J mice from the Exercise Experiment (with or without wheels) that has at least one myofiber containing (A and F) areas of regeneration, (B and G) perimysial infiltration, (C and H) pale staining cytoplasm, (D and I) cellular invasion, and (E and J) necrotic fibers. Values are percentage of the population. DG = deep gastrocnemius, SG = superficial gastrocnemius, PL = plantaris, SL = soleus.

Overall, the superficial gastrocnemius had relatively fewer areas of regeneration than did the other muscles, whereas the soleus had relatively more perimysial infiltration and more necrotic fibers that the other muscles (Fig 6A–6E). Similar patterns were seen for areas of regeneration and necrotic fibers when comparing mini- and normal-muscled individuals (Fig 6F–6J).

## Discussion

### Effects of contusion injury on voluntary wheel-running behavior

Our protocol for contusion injury was similar to those used in previous studies that have shown negative effects on muscle function [5,43], so we presumed a priori that running ability would be reduced in the present study. Interestingly, contusion injury decreased wheel running only in mice from HR lines (Fig 2). This result suggests that, under normal conditions, the ability of C mice to run on wheels exceeds their motivation and that the amount of injury experienced did not decrease this ability to a level that would reduce their daily wheel running below the amount dictated by their inherent motivation to run voluntarily on wheels. For uninjured HR mice, the amount they are able to run is probably similar to the amount they are motivated to run, given that they have been under continued selection for tens of generations since they reached selection limits [37], such that any reduction in either ability or motivation will cause a reduction in daily running distance.

Hypothetically, changes in mean (or maximum) running speed—which mostly account for the higher daily running distances by HR mice—are closely aligned with changes in running ability, whereas changes in duration of running may be more reflective of motivation. In our study, contusion injury reduced running speeds of HR mice, but not their daily running duration (Fig 2), which suggests negative effects on ability but not motivation. If this interpretation is correct, then it is somewhat surprising because contusion injury typically leads to pain during movement [e.g., see 4], which could negatively affect motivation [e.g., see discussion in 50].

In principle, HR mice might have evolved reduced pain sensitivity or increased pain tolerance. A study of opioid-mediated pain sensitivity did not find statistically significant differences between female HR and C mice [51]. However, endocannabinoids also modulate pain both peripherally and centrally in rats and humans [52,53], and pharmacological studies show differences in endocannabinoid function between HR and C mice of both sexes [54] and that 6 days of wheel access differentially affects circulating concentrations of anandamide in both sexes [42]. Another study showed that HR female mice tended to have relatively larger periaqueductal grey (PAG; an area of the midbrain associated with pain perception) volumes than C females when housed without wheels, but this difference was reversed when mice were housed with wheels for 10 weeks beginning at weaning [55]. Initial differences or changes in PAG volume or plasma anandamide levels during 6 days of wheel running could lead to reduced pain sensitivity or increased pain tolerance in HR mice, helping them to recover motivation for running after an injury.

On day 1 following injury, injured HR mice ran ~38% fewer revolutions/day (Fig 2A). Both injured and uninjured HR mice increased daily revolutions run across the following five days, but on day 6 the injured animals still ran ~21% fewer revolutions than uninjured HR mice. Thus, recovery from injury was not complete by day 6. Exercise is one of several factors than can affect muscle healing from contusion injury [see 4 for review]. Rats have shown an increase in muscle repair after contusion injury to the gastrocnemius when given access to wheels (one hour/day) for three days, and have shown a more complete response to repair after 21 days with wheel access than immobilized controls [3]. In mice, treadmill running (one hour/day, five days/week for five weeks) reduces the amount of collagen observed as a result of a contusion injury to the tibialis anterior [56]. Mice also show increased satellite cell activity if allowed to voluntarily run on wheels during recovery from hindlimb suspension unloading [57]. To our knowledge, no other published study has given animals access to wheels *ad libitum* following contusion injury.

### Exercise-induced injury

#### Plasma creatine kinase activity

Contrary to our hypothesis, voluntary wheel running for 6 days had no statistically significant effect on plasma CK levels, nor did linetype. To our knowledge, no previous study has examined effects of voluntary wheel running on plasma creatine kinase levels in such a short time scale. However, a study that used 3 weeks of wheel access and sampled blood one day after the end of the wheel access found no effect on serum creatine kinase [58]. Strenuous exercise has been shown to increase circulating CK levels in rodents [8,treadmills: 13,58] and in humans [59–63]. Also, human athletes (both strength and endurance) have higher resting plasma CK levels when compared to sedentary/non-athletic controls [64–66], which is probably caused by increased training. For example, Chevion et al. [67] showed that individuals that routinely experience high-volume intense exercise have higher baseline levels of serum CK than untrained or more moderately trained individuals. Moderate exercise may not induce changes in membrane permeability and, therefore, no increase in serum CK levels should be expected unless training/exercise exceeds this threshold [68–71]. The amount of wheel exercise performed by C and even HR mice may not be sufficient to elicit changes in membrane permeability that would cause a significant increase in plasma CK levels: even HR mice rarely reach their maximal aerobic speed during voluntary wheel running [15,72]. Another possible explanation for the similarity in plasma CK levels between HR and C mice after wheel access is that the former have altered stride characteristics [25], which might decrease sarcolemmal disruption, decreasing the leakage of CK from the muscle.

Although we did not find differences in plasma CK activity between HR and C mice or between exercised and non-exercised individuals, mini-muscle mice had lower plasma CK activity. Yamashita and Yoshioka [73] showed that total CK is more prevalent in fast-twitch glycolytic fibers (MyHC-2b), lower in MyHC-2a, and lowest in MyHC-1. Therefore, the lower plasma CK levels of mini-muscle mice probably reflect their reduced numbers of MyHC-2b fibers [28,32]. On the other hand, mini-muscle mice have ~1/3 of the compartment PCSA, so a given cross-sectional area of muscle should experience significantly greater stresses than comparable muscles in non-mini-muscle mice. Therefore, one would expect greater stretch-induced damage in mini-muscles. Plasma CK levels will reflect a balance between these two factors.

The utility of plasma CK activity as a marker for gauging muscle injury has been debated [e.g., see 74,75]. We therefore tested for correlations between plasma CK activity and the histological measures of muscle injury in each of the four muscle areas across all of our samples (all of the histological measures were log-transformed). Only one of the 20 possible correlations reached statistical significance, and it was in the opposing direction (CK versus % of central nuclei in the superficial gastrocnemius: N = 91, r = -0.214, P = 0.041). Results from the present study suggest that plasma CK is limited in its usefulness as a marker of muscle injury.

#### Central nuclei

Central nuclei occur during the regenerative processes that follows muscle injury. During this process, new myogenic cells fuse to form myofibers with centrally located nuclei, which can be quantified as an indicator of regeneration. The nuclei then migrate to the periphery as the regenerative process is completed [see 76 for review]. In the soleus, HR mice had a higher percentage of fibers with central nuclei than C mice, with no statistical interactions, indicating that HR mice are not more resistant to exercise-induced injury than C mice. Similar results were not seen in other muscles of the triceps surae complex (Table 4). The plantaris tended to have a higher percentage of central nuclei in mice that had 6 days of wheel access (P = 0.0525; Table 4). The fibers in the plantaris of HR mice had a significantly increased percentage of central nuclei when the total number of revolutions or speed of running was added to the model, and both of these were negatively predictive of central nuclei. One possible explanation for this observation is that as little as a single bout of (forced treadmill) exercise has been shown to have a protective effect against muscle injury [8]. Also, wheel running itself may speed muscle recovery [3], and the regenerative processes can start as early as 96 hours post-injury in rodents [13,77,78]. Thus, fibers in the plantaris could have been injured during the early period of wheel access (e.g., during day 1 or 2), but somewhat protected from further injury, and also the subsequent wheel running could have facilitated muscle recovery. This scenario could explain the abundance of central nuclei in HR mice (with wheel running as a covariate) in the plantaris, as well as the negative relationship with total revolutions or speed.

In addition to the effects of linetype and wheel access, mini-muscle individuals had more central nuclei in the deep and superficial gastrocnemius and the plantaris, and wheel access increased this in superficial gastrocnemius while decreasing the effect in soleus (mini*wheel interaction: Table 4, Fig 4B). We do not believe that the increased number of central nuclei in the superficial gastrocnemius of mini-muscle mice is related to injury, but to their muscle fiber phenotype in general. Talmadge et al. [28] noted the increased number of MyHC-2b fibers containing central nuclei in the superficial region of the gastrocnemius of untrained adult mini-muscle mice compared to other untrained HR or C57Bl6NHsd mice. Because some studies have shown that oxidative fibers are more likely to be injured due to (forced treadmill) exercise [8,13,79,80], the increase in the percentage of central nuclei in the deep gastrocnemius and the plantaris may be explained by the higher percentage of type MyHC-1 and MyHC-2a fibers that exist in these areas in mini-muscle mice compared to normal-muscled mice.

#### Total muscle injury and regeneration

Most mice and most muscle fibers did not show signs of injury; however, mice with wheel access showed significantly higher levels of injury in the soleus and higher levels of regeneration in the plantaris, for both HR and C mice (Fig 6). Komulainen and Vihko [81], subjected male rats to exhaustive exercise on an inclined treadmill and fiber swelling and interstitial edema was observed in the soleus 4–12 hours post-exhaustion, with histological markers indicative of muscle injury (e.g., inflammation, necrosis) seen 12–96 hours post-exhaustion, depending on the muscle in question. In rats, muscle injury occurs earlier in the plantaris than the soleus [82]. Thus, in the present study, the plantaris may have been injured by wheel running earlier than the soleus, and then began regenerating while the soleus was still in the injury phase.

Mini-muscle mice had higher levels of injury and regeneration in the deep and superficial regions of the gastrocnemius (Table 4, Fig 6). Total regeneration is a function of the percentage of central nuclei and number of areas of regeneration seen in a single muscle. The superficial gastrocnemius of mini-muscle mice have inherently more central nuclei, which accounts for their higher total regeneration and may not be indicative of injury [28; see Fig 4B]. However, the increased regeneration in the deep gastrocnemius is a relatively equal combination of areas of regeneration and central nuclei. The injury in the deep and superficial gastrocnemius is a combination of perimysial infiltration, invaded cells, pale cytoplasm, and increased necrotic fibers in mice with wheel access (Fig 6). The superficial gastrocnemius is comprised almost entirely of MyHC-2b muscle fibers (with a smaller amount present in the deep gastrocnemius) in normal-muscled individuals which are able to handle higher forces than other fiber types. Mini-muscle mice have far fewer MyHC-2b fibers, which may explain the increase in injury/recovery seen in the superficial (and deep) gastrocnemius.

Within mini-muscle mice, the soleus and plantaris showed a mini*wheel access interaction for regeneration: with wheels, regenerating fibers increased for the plantaris, but decreased for the soleus (Fig 6). This is possibly due to the different time courses for injury and regeneration in the two muscles [82]. Another explanation are findings that show the plantaris is less resistant to injury than the soleus [10,83]. The authors claim that this is due to the plantaris having a higher proportion of MyHC-2 fibers, but this is contradictory to findings of other authors who state that MyHC-2 fibers are more resistant to injury [8,e.g., 13,79,80].

### Comparisons of different muscle groups

In the superficial gastrocnemius, fewer individuals had areas of regeneration than for any other muscle (Fig 6A). The soleus had an increased proportion of necrotic fibers and increased perimysial infiltration compared with other muscles. In general, we observed fewer signs of injury and areas of regeneration (but not central nuclei) in the superficial gastrocnemius, which may reflect the increased force potential of its many MyHC-2b fibers and their lower propensity to experience exercise-induced injury (see references in Section *4*.*3 Exercise-Induced Injury*: *Central Nuclei*, although these studies used treadmill running for the induction of injury). Additionally, exhaustive uphill running (concentric exercise) may preferentially injure type I fibers, which are lacking in the superficial gastrocnemius [84].

### Conclusions, limitations, and future directions

Contrary to our initial hypotheses, our results suggest that HR mice have not evolved a heightened ability to resist muscle injury (exercise experiment), nor do they recover from injury faster than mice from non-selected C lines (contusion experiment). Within the HR lines of mice, individuals with the mini-muscle phenotype have more indicators of both injury and regeneration than normal-muscled mice, even when they do not have access to wheels, which is likely related to their reduced numbers of MyHC-2b muscle fibers. This finding does not support our initial hypothesis that only the superficial gastrocnemius of mini-muscle mice would show increased muscle injury/regeneration compared to normal-muscled individuals, or that the other muscles of the triceps surae complex of mini-muscle mice would show fewer signs of injury or regeneration. Finally, the number of injured or regenerating fibers was very small, even for HR mice given six days of wheel access; thus, increased amounts or intensities of running, possibly by use of forced treadmill exercise, may be required to show a substantial increase in injured or regenerating fibers in these mice. Greater amounts of injury caused by longer durations of wheel running or more extensive contusions might also reveal differences between HR and C mice.

This study is somewhat limited by the absence of video recording. We are unable to determine the quantity of up- versus downhill running performed by each mouse. A relatively greater amount of downhill running would increase the number of lengthening contractions, and thus the amount of injury in a given mouse. However, the purpose of this study was to see if wheel running, in general, caused more or less injury in mice specifically bred for wheel running, regardless of the intensity or time spent running up- or downhill. Additionally, this study is limited by its use of only H&E staining without the use of fiber-type or other antibody staining to mark injured muscle fibers. Some directions for future studies would be to examine fiber type, physiological cross-sectional area, fiber swelling, collagen infiltration (via Masson’s trichrome staining), immunostaining for dystrophin, time courses of injury and recovery, the use of previously trained individuals, and, as mentioned above, longer durations of wheel running. Other future studies could use an *in-situ* muscle preparation to determine if contusion or wheel running causes decreased muscle performance per se in HR vs. C mice.
